# Supplementary material for: Causal Relationship between Aging and Anorexia Nervosa: A White-Matter-Microstructure-Mediated Mendelian Randomization Analysis
Source: Biomedicines. 2024 Aug 16;12(8):1874. doi: 10.3390/biomedicines12081874 (PMC11351342; doi:10.3390/biomedicines12081874)
Supplement: Supplementary file 1 [file biomedicines-12-01874-s001.zip › Supplementary Figure/Figure S8/Leaveoneout plot of MR analysis for TL to ubm-b-1591.pdf]

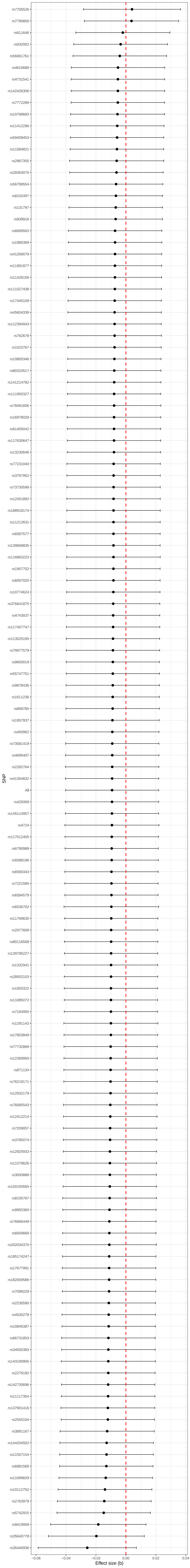

rs7705526
rs7790856
rs611646
rs932002
rs56061761
rs4616688
rs4731541
rs142426306
rs7772289
rs10768683
rs11412296
rs59409453
rs11584821
rs2967355
rs28363070
rs56799554
rs8102497
rs131797
rs939916
rs6669563
rs1985369
rs41269079
rs11991877
rs11426156
rs111527438
rs17445108
rs45604339
rs112394943
rs762679
rs1023767
rs10805346
rs80324517
rs141214782
rs111950327
rs78491606
rs16978028
rs61405042
rs117630647
rs13230646
rs77231040
rs3767952
rs73730598
rs12451892
rs188918174
rs11212631
rs6587577
rs139669835
rs116863223
rs1907702
rs6007020
rs10774624
rs376641875
rs4743037
rs117407747
rs113525195
rs79977579
rs9600019
rs55747751
rs9878436
rs1611236
rs869785
rs1957937
rs450962
rs73581419
rs4695407
rs2282764
rs41304832
All
rs429358
rs145114957
rs4724
rs117512405
rs6790988
rs9398196
rs6590343
rs7221585
rs6584579
rs6536702
rs11769630
rs2977608
rs80116508
rs139795227
rs1332941
rs28502153
rs1003322
rs11085072
rs7164950
rs1291143
rs17803849
rs77732866
rs12369950
rs871134
rs76219171
rs12932179
rs76065543
rs12412214
rs7209057
rs3785074
rs12925933
rs11579626
rs3093888
rs150150565
rs8105767
rs9955360
rs76666449
rs6659669
rs202034370
rs185174247
rs17677991
rs182059586
rs7099229
rs2230590
rs4530278
rs10845387
rs66731853
rs34550383
rs143190905
rs2276182
rs142730696
rs11117354
rs137901416
rs2555104
rs3891167
rs144204502
rs11557154
rs6881568
rs11699829
rs10112752
rs2763979
rs5742915
rs9419958
rs35640778
rs35446936
SNP
-0.06
-0.04
-0.02
0.00
0.02
0.04
Effect size (b)
